# Evaluation of cost-effective total nucleic acids extraction protocols for cultured *Mycobacterium tuberculosis*; a comparison by PCR amplification of genes associated with drug resistance

**DOI:** 10.1186/1756-0500-3-48

**Published:** 2010-02-26

**Authors:** Adolf K Awua, Edna D Doe, Oti K Gyamfi

**Affiliations:** 1 Cellular and Clinical Research Centre, Radiological and Medical Sciences Research Institute, Ghana Atomic Energy Commission, P.O. Box LG 80, Legon-Accra, Ghana

## Abstract

**Background:**

The emergence of drug resistant strains of *Mycobacterium tuberculosis *complex has made the management of tuberculosis difficult. Also, *Mycobacterium species *has a peculiar cell wall, made of an impermeable complex structure rich in mycolate, making the lyses of its cell difficult. In order to apply a radio-labelled-probe based detection of mutations in selected genes leading to drug resistance, we concede that the evaluation and modifications of nucleic acid extraction protocols that are less sophisticated and less prone to contamination would be useful in the management of tuberculosis in a resource-constrained setting.

**Findings:**

The average amount of nucleic acids was determined for different extraction treatments. High temperature treatment only, yielded the lowest amount of nucleic acids, i.e. 15.7 ± 3.2 μg. The average amount of nucleic acids obtained with the addition of TE and triton-X100, was 133.7 ± 8.9 μg, while that obtained with the addition of TE only, and TE and SDS were 68.4 ± 22.7 μg and 70.4 ± 20.3 μg respectively. Other treatments yielded 28.8 ± 6.7 μg, 32.5 ± 2.4 μg and 36.9 ± 15.5 μg. The average amount of nucleic acids obtained with high temperature treatment in TE, and that obtained by freezing prior to high temperature treatment, successfully amplified for the genes of interest (*rpoB, KatG, rrs*).

**Conclusion:**

We strongly recommend the use of 1× TE buffer, and freezing and heating for improved lysis of cultured *M. tuberculosis*, and therefore, as an effective method for the preparation of *M. tuberculosis *nucleic acid useful for PCR.

## Findings

### Justification

The application of most molecular biology techniques is largely dependent on the quantity and quality of extracted nucleic acids [1,2] and which are, in turn, greatly influenced by the efficiency of cell lysis, DNA/RNA recovery and residual amounts of some extraction reagents [3-5].

*Mycobacterium *cell wall is made of an impermeable complex structure that makes the lyses of the cell difficult [3,6,7]. As a result, most of the simple and commonly used nucleic acid isolation procedures result in poor quality and low yield of nucleic acids, and are also affected by the type of specimen used [7-9]. Several complicated protocols and commercial DNA and RNA extraction kits have been developed that are mostly efficient on cultured *Mycobacterium*, but these are expensive, and have elaborate procedures [10,11].

PCR amplifications, in our laboratory, of some of the resistance-associated genes of the *Mycobacterium tuberculosis-*complex genome have not been appropriate for further analysis. A number of PCR protocol modifications resulted in marginal improvement of the amplification of these genes. Therefore, we concede that the evaluation and modifications of nucleic acid extraction protocols that are less expensive, less sophisticated, and less prone to contamination would be useful in resource-constrained setting as in Ghana. We therefore present a comparison of the results of seven nucleic acids extraction protocols, and aim at determining a cost-effective and less time consuming extraction protocol for cultured *Mycobacterium tuberculosis *by PCR of drug resistance genes.

## Methods

### Culture and Cell Suspension Preparation

Colonies, from five sputum samples cultured on (Lowenstein Jensen) medium slants for seven weeks, were harvested to prepare a two loopfuls of colony per mL suspension in BBL™ Middlebrook ADC Enrichment broth.

For each DNA extraction protocol, an aliquot (200 μL) of each cell suspension was transferred into a sterile 1.5 mL screw-capped tube and centrifuged for 3 min at 10,000 × g. The supernatant was carefully removed and the pellet washed twice with sterile distilled water, centrifuging each time as above. The five resultant pellets were then subjected to total nucleic acid extraction.

### Nucleic Acid Extraction

#### Protocol 1

The resultant pellets were carefully re-suspended in 200 μL of sterile distilled water, mixed by vortex, and placed on a heating block at 95°C for 30 min. After cooling at room temperature and centrifuging at 5000 × g for 10 min; the supernatants were transferred to a new sterile 1.5 mL tube and stored at 4°C.

#### Protocol 2

A suspension of each pellet in 200 μL of sterile distilled water was mixed by vortex. To this, 22.2 μL of 10% SDS was added and mixed gently by inversion, and thereafter incubated on a heating block at 65°C for 30 min. The resultant lysate was allowed to cool to room temperature. Subsequent to lysate incubation on ice for 10 min and centrifugation at 5000 × g for 10 min, the supernatant was recovered to a new 1.5 mL tube. The supernatant was incubated with 2 volumes of absolute ethanol on ice for at least 1 hour, and centrifuged at 12,000 × g for 10 min; the resultant supernatant was decanted and pellets air dried. The pellets were mixed gently in 50 μL of warm sterile distilled water and stored at 4°C.

#### Protocol 3

Cell pellets suspended in 200 μL of sterile distilled water, were frozen at -20°C. Upon thawing on ice, the suspensions were incubated at 100°C for 30 min. Lysates were mixed and centrifuged at 6000 × g for 10 min. The supernatants were recovered to a new 1.5 mL tube and stored at 4°C.

#### Protocol 4

Cell deposits were incubated at 95°C for 30 min as a suspension in 200 μL of lysis buffer (10 mM Tris-HCl and 1 mM EDTA) and the resulting lysates were gently mixed and centrifuged at 5,000 × g for 10 min. The supernatants were recovered and stored at 4°C.

#### Protocol 5

Cell pellets in 200 μL of lysis buffer (10 mM Tris-HCL and 1 mM EDTA), were mixed gently with 22.2 μL of 10% SDS by inversion, and incubated at 65°C for 30 min. The lysates were allowed to cool to room temperature before incubating on ice for 10 min. Following centrifugation at 5,000 × g for 10 min, recovered supernatants were then incubated with 2 volumes of absolute ethanol on ice for at least 1 hour. DNA was recovered by centrifugation at 12,000 × g for 10 min, air-drying pellet, and re-suspending pellet in 50 μL of warm 1× TE buffer. These were stored at 4°C.

#### Protocol 6

Aliquots (150 μL) of lysis buffer (10 mM TRIS-HCl pH 8/1 mM EDTA/1% Triton-X 100) were added to cell pellets and incubated 95°C for 30 min. Resultant lysates were then mixed by vortex and centrifuged for 8 min at 7,000 × g. Subsequently, supernatants were carefully recovered and stored at 4°C.

#### Protocol 7

This was a repeat of protocol 6 above with 1% Tween-20 replacing TritonX-100.

### PCR amplification of Mycobacterium tuberculosis DNA

Five microlitres (5.0 μL) of each extract was used with Qiagen PCR kit for the gene amplification. Commercially purified DNA of *Mycobacterium tuberculosis *H_37_RV standard strain and PCR water were used as positive and negative controls respectively. The following primers were used;

#### rpoB gene

(*rpoB*For; TGGTCC GCTTGC ACGAGG GTCAGA and *rpoB*Rev; CTCAGG GGTTTC GATCGG GCACAT).

#### KatG gene

(RTB39; TGGCCG CGGCGG TCGACA TT and RTB38; GGTCAG TGGCCA GCATCG TC).

#### rrs gene

(STR53; TCACCA TCGACG AAGCTC CG and STR31; CTAGAC GCGTCC TGTGCA TG).

#### rpsL gene

(STR52; GTGAAG ACCGCG GCTCTG AA and STR34; TTCTTG ACACCC TGCGTA TC).

The cycling programme was: initial denaturation at 95°C for 15 min; followed by 45 cycles of denaturation at 94°C for 1 min, annealing 60 - 64°C for 1 min and an extension at 72°C for 1 min; a final extension step at 72°C for 10 min was performed.

### Electrophoresis

Amplicons were resolved on an ethidium bromide stained 2% agarose gel.

In avoiding contaminations, culture processing was performed in a separate biosafety cabinet located in separate room; nucleic acids extracts and PCR mixes were prepared in PCR work stations in separate rooms; and disposable plugged micropipette tips were used.

### Relative Quantification of Nucleic Acids

Extracts obtained by each protocol was incubated with 2 volumes of absolute ethanol on ice for at least 1 hour and centrifuged at 12,000 × g for 10 min. Supernatant were decanted and pellets washed with 70% ethanol. The centrifugation was repeated; the pellets were air-dried and resuspended in 50 μL of warm 1× TE buffer.

The yield of total nucleic acid for each protocol was determined by measuring and calculating the average absorbance at 260 nm of the five extracts. Also, absorbance at 280 nm was measured and the ratio of UV absorbance 260/280 nm calculated.

## Results

### Growth from Culture

Rough, eugonic buff colonies were first observed for all the five cultured sputum samples, three weeks after incubation, and stained acid-fast. At harvest, growth covered at least 50% of the media.

### Nucleic Acids Quantity

The average amount of nucleic acids was determined for each protocol and presented in table 1. Protocol 1 yielded the lowest amount of nucleic acids i.e. 15.7 ± 3.2 μg. This is due to poor lysis, achieved by only heating in sterile distilled water. The yield doubled when the cell suspension was frozen, and thawed at 0°C prior to heating (protocol 3). These indicate that the heat shock process improved cell lysis by a great extent (table 1). The use of SDS at a final concentration of 1% in addition to heating the cell suspension as described in protocol 2, also doubled the yield of nucleic acids (table 1). It is therefore evident from comparing the results of protocols 2 and 1 that the use of SDS at 1% improved the effectiveness of cell lysis. However, upon comparing the results of protocols 2 and 3 (table 1), it can be noticed that the effectiveness of SDS at improving cell lysis was achieved by freezing the cell suspension prior to heating.

**Table 1:** Quantity of nucleic acids extracts obtained by the respective protocols

	Protocol 1	Protocol 2	Protocol 3	Protocol 4	Protocol 5	Protocol 6	Protocol 7
**Relative amount of nucleic acids**	1	~2	>2	4	4	8	2

**Amount of nucleic acid (μg)**	15.7 ± 3.2	28.8 ± 6.7	36.9 ± 15.5	68.4 ± 22.7	70.4 ± 20.3	133.7 ± 8.9	32.5 ± 2.4

**Temp' Treatments**	heating	heating	freezing and heating	heating	heating	heating	heating

**Chemical Treatments**		SDS		Tris/EDTA	Tris/EDTA/SDS	Tris/EDTA/Triton X-100	Tris/EDTA/Tween 20

**Ratio of UV absorbance (A_260/280 nm_)**	0.20	1.18	1.23	1.25	1.39	0.73	1.27

Protocol 4 recorded a four fold increase in the yield of nucleic acids relative to that obtained with protocol 1 (table 1). This can be attributed to the fact that cells of *Mycobacterium *were better dispersed in the 1× TE buffer as used in protocol 4 [3]. Therefore, a comparison of the yield obtained for protocol 4 with those for protocols 2 and 3 (table 1) reveals that the inclusion of Tris-EDTA led to a more efficient lysis compared to the use of SDS alone at 1% (protocol 2), as well as freezing prior to heating (protocol 3). As presented in table 1, the yield recorded with protocol 5 (which involved the use of Tris-EDTA, SDS and heat) was not significantly different from that recorded by protocol 4 (which involved the use 1× Tris-EDTA and heat). This indicates that the inclusion of SDS in protocol 5 did little to improve the efficiency of lysis when it was used with Tris-EDTA. Coupled with the earlier finding that the efficiency of SDS (in protocol 2) could be achieved by freezing and heating (protocol 3), and also by heating in TE (protocol 4), the use of SDS at 1% could be conveniently avoided on the bases of extra cost [5].

Using TritonX-100 with Tris-EDTA (Protocol 6) clearly improved the efficiency of lysis; since the yield of nucleic acids was eight times that obtained with protocol 1 and four times that obtained with protocol 4. TritonX-100 is known to prevent the aggregation of *Mycobacteria *in suspension, thereby, enhancing the effect of heating on the cell wall structure [3]. The use of Tween-20 in addition to TE with heating (protocol 7) recorded a yield about half that obtained with protocol 4, (table 1).

### Amplification by PCR

**Figure 1:**
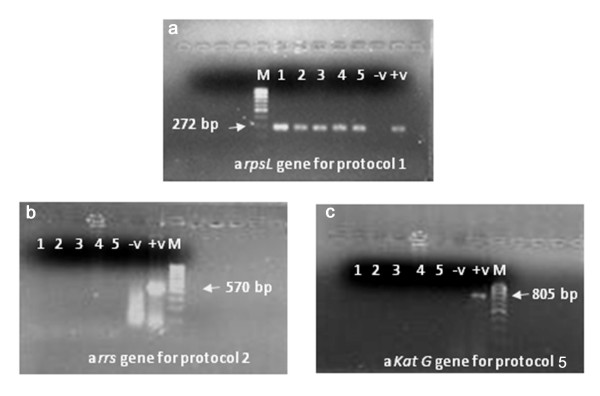
**Amplification products for protocol 1, 2 and 5 respectively**. a) *rspL *b) *rrs *c) *KatG *genes DNA extracted from five cell suspensions with each of the protocols 1, 2 and 5 were amplified by PCR for three genes. Amplification products of the *rpoB, KatG and rrs *genes obtained were each resolved at 70 Volts for 1 hour on a 2% agarose mini-gel containing 0.001 mg/mL ethidium bromide.

**Figure 2:**
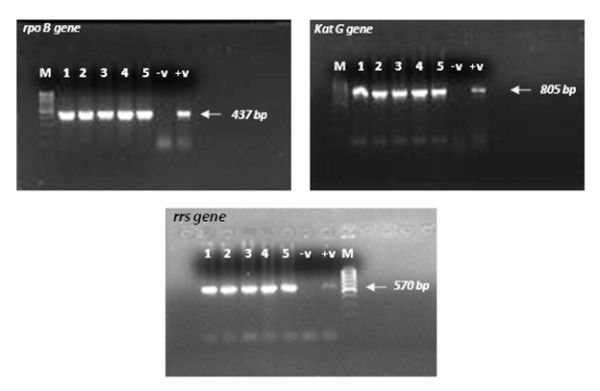
***rpoB*, *KatG *and *rrs *gene amplifications product of DNA obtained with protocol 4**. DNA extracted from five cell suspensions with protocol 4 were amplified separately by PCR for three genes. Amplification products of the *rpoB, KatG and rrs *genes obtained separately by PCR were each resolved at 70 Volts for 1 hour on a 2% agarose mini-gel containing 0.001 mg/mL ethidium bromide.

**Figure 3:**
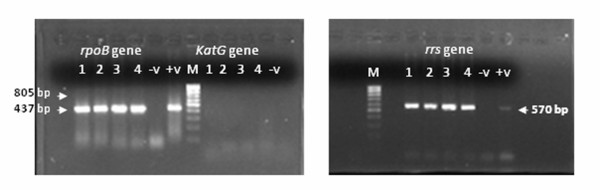
***rpoB*, *KatG *and *rrs *gene amplifications product of DNA obtained with protocol 3**. DNA extracted from five cell suspensions with protocol 3 were amplified by PCR for three genes. Amplification products of the *rpoB, KatG and rrs *genes obtained were each resolved at 70 Volts for 1 hour on a 2% agarose mini-gel containing 0.001 mg/mL ethidium bromide.

The difficulties we have had with the amplification of the *KatG *and *rpoB *genes using extracts obtained by protocol 1 were again observed in this study. This inconsistent amplification is likely to be a result of the low yield and/or poor quality of DNA, although the *rpsL *gene amplified for this extract (figure 1). Poor quality DNA is most likely the cause of this observation since the amount of nucleic acid did not correlate with the PCR amplification and also that the ratio of UV absorbance at260/280 nm was 0.2 (table 1), indicating the extract contained a relatively high amount of protein, which could inhibit PCR [4]. Extracts obtained with protocol 4 amplified for all three genes (table 2, *rpoB, KatG *and *rrs*), showing as bright bands in figure 2. Clearly, the higher nucleic acids yield and ratio of UV absorbance obtained by protocol 4 relative to protocol 1 influenced the successful amplifications since PCR is known to be influenced by the amount of nucleic acids and proteins amongst other factors [4,5]. Interestingly, extracts obtained by protocol 3 amplified for two of the three genes (*rpoB *and *rrs*; table 2 and figure 3), and recorded yields and ratio of UV absorbance higher than those obtained with protocol 1, but lower than those obtained with protocol 4 (table 1). The non-amplification observed for extracts obtained with protocols 2 and 5 (figure 1b and 1c) is likely to be due to residual SDS (a known inhibitor of PCR) in the extracts; since their nucleic acids yields and UV absorbance ratios were higher than for other extracts that amplified by PCR (table 1). The use of SDS for the extraction of *Mycobacterium *DNA would require further purification steps in other to fully take advantage of the lysis efficiency of SDS. PCR amplification of DNA obtained with protocols 6 and 7 were not consistent and reproducible. This observation remains unclear, since the yields were quite high.

**Table 2:** Amplification of selected genes for each the seven protocols evaluated

Genes amplified	Protocol 1	Protocol 2	Protocol 3	Protocol 4	Protocol 5	Protocol 6	Protocol 7
*rpo B*	X	X	√	√	X	-	-

*Kat G*	X	X	X	√	X	-	-

*rrs*	√	X	√	√	X	-	-

**Treatments**							

*Temperature*	heating	heating	freezing and heating	heating	heating	heating	heating

*Chemical*		SDS		Tris/EDTA	Tris/EDTA/SDS	Tris/EDTA/TritonX-100	Tris/EDTA/Tween20

√ ⇒ expected amplification; X ⇒ no amplification; - ⇒ inconsistent amplification

We strongly suggest the use of 1× TE buffer in addition to temperature treatments for improved lysis of *M. tuberculosis *form culture. A combination of freezing and heating in Tris-EDTA (protocols 3 and 4) is strongly recommended for the preparation of *M. tuberculosis *nucleic acids useful for PCR; since in addition to its effectiveness, the reagents used in the two protocols are easily available.

## Competing interests

The authors declare that they have no competing interests.

## Authors' contributions

AKA and EDD were involved in the designed of the study and were also responsible for the laboratory analyses, while OKG designed protocol 6. AKA wrote the initial draft and all the authors read and approved the final manuscript.

## References

[B1] Bachmann L Däubl B Lindqvist C Kruckenhauser L Teschler-Nicola M Haring E PCR diagnostics of *Mycobacterium tuberculosis *in historic human long bone remains from 18th century burials in Kaiserebersdorf, Austria BMC Research Notes 2008 1 83 10.1186/1756-0500-1-83 18799009 PMC2556691

[B2] Taylor GM Worth DR Palmer S Jahans K Hewinson RG Rapid detection of *Mycobacterium bovis *DNA in cattle lymph nodes with visible lesions using PCR BMC Veterinary Research 2007 3 12 10.1186/1746-6148-3-12 17567891 PMC1904440

[B3] Kotlowski R Martin A Ablordey A Chemlal K Fonteyne P-A Portaels F One-tube cell lysis and DNA extraction procedure for PCR-based detection of *Mycobacterium ulcerans *in aquatic insects, molluscs and fish J Med Microbio 2004 53 927 933 10.1099/jmm.0.45593-0 15314202

[B4] Honore'-Bouakline S Vincensini JP Giacuzzo V Lagrange PH Herrmann JL Rapid Diagnosis of Extrapulmonary Tuberculosis by PCR: Impact of Sample Preparation and DNA Extraction J Clin Microbiol 2003 2323 2329 10.1128/JCM.41.6.2323-2329.2003 12791844 PMC156509

[B5] Amita J Vandana T Guleria RS Verma RK Qualitative Evaluation of Mycobacterial DNA Extraction Protocols for Polymerase Chain Reaction Mol Biol Today 2002 3 43 50

[B6] Reischl U Application of molecular biology-based methods to the Diagnosis of infectious diseases Frontiers in Bioscience 1996 1 e72 77 9159247 10.2741/a145

[B7] Barry MC Mdluli K Drug sensitivity and environmental adaptation of mycobacterial cell wall components Trends Microbiol 1996 4 275 8 10.1016/0966-842X(96)10031-7 8829336

[B8] Kolk AHJ Schuitema ARJ Kuijper S Van Leeuwen J Hermans PWM Van Embden JDA Hartskeerl RA Detection of Mycobacterium tuberculosis in clinical samples by using polymerase chain reaction and a non radioactive detection system J Clin Microbiol 1992 30 2567 2575 1400955 10.1128/jcm.30.10.2567-2575.1992 PMC270480

[B9] Fries JWU Patel RJ Piessehn WF Wirth DE Detection of untreated *Mycobacteria *by using polymerase chain reaction and specific DNA probes J Clin Microbiol 1991 29 1744 1747 1761699 10.1128/jcm.29.8.1744-1747.1991 PMC270197

[B10] Boddinghaus I Rogall T Flohr T Blocker H Bottger EC Detection and identification of Mycobactera by amplification of rRNA J Clin Microbiol 1990 28 1751 1759 2203812 10.1128/jcm.28.8.1751-1759.1990 PMC268042

[B11] Alvarado-Esquivel C García-Corral N Carrero-Dominguez D Enciso-Moreno JA Gurrola-Morales T Portillo-Gómez L Rossau R Mijs W Molecular analysis of *Mycobacterium *isolates from extrapulmonary specimens obtained from patients in Mexico BMC Clin Patho 2009 9 1 10.1186/1472-6890-9-1 PMC2660362 19272158

